# Development of a New Measure of Intergenerational Contact

**DOI:** 10.1093/geroni/igab046.2906

**Published:** 2021-12-17

**Authors:** Karen Hooker, Shelbie Turner, Shannon Jarrott, John Geldhof

**Affiliations:** 1 Oregon State University, Corvallis, Oregon, United States; 2 Oregon State University, Oregon State University, Oregon, United States; 3 The Ohio State University, Columbus, Ohio, United States

## Abstract

Intergenerational relationships are a predictor of greater physical, mental, and emotional well-being; they can reduce ageism and facilitate feelings of purpose and meaning, shown to be important for health and mortality. Surprisingly there are no measures of intergenerational contact (IGC) shown to be reliable and valid across age groups. Therefore, we aimed to develop a psychometrically sound survey measure of IGC. We utilized a three-phase development process, which included a Delphi-style expert panel review of items, focus groups, and validation of the survey via Amazon MTurk. The resulting 18-item survey captures details on and comparisons between both familial and non-familial contact and is appropriate for adults of all ages. We conducted confirmatory factor analyses with the 9-item family and 9-item non-family subscales for 380 young and 256 middle-aged adults reporting on contact with older adults, and 348 older adults reporting on contact with younger adults. The family scale had good model fit across all three groups (χ2 (78) = 245.74, p<.0001; RMSEA = 0.08, 90% CI = [0.07, 0.10]; CFI = .94; TLI = .92). After covarying for 2 pairs of items among the middle-age group, the non-family scale had good model fit across all groups (χ2(75) = 217.21, p<.0001; RMSEA = 0.08, 90% CI = [0.07, 0.09]; CFI = .95; TLI = .93), indicating construct equivalence across age groups. The new IGC measure can be used in all adult age groups, making it useful for scientific projects as well as program evaluations. Funded by the RRF Foundation on Aging

